# Otomastoidititis with right retroauricular fistula by Lagochilascaris minor

**DOI:** 10.1590/S1808-86942010000300025

**Published:** 2015-10-20

**Authors:** Roig O.R. Jorge Luis, José Luis Roig-Ocampos Forteza, Lidio Granato, Daniel Poletti Serafini

**Affiliations:** 1 Professor, PhD in Medicine, Associated physician – Otolaryngology Department – University Hospital of Universidade Nacional de Asunción – Paraguay; 2 Professor, PhD in Medicine, Head of the Otolaryngology and Head and Neck Surgery – University Hospital – Universidade Nacional de Asunción; 3 Professor, PhD in Medicine, Assistant Professor – Santa Casa de Misericórdia de São Paulo; 4 PhD in Medicine, Resident Physician – General University Hospital Gregorio Marañon, Madrid. Hospital de Clínicas de la Facultad de Ciencias Médicas de la Universidad Nacional de Asunción

**Keywords:** parasitic diseases, cutaneous fistula, mastoiditis, otitis media

## INTRODUCTION

Lagochilascaris minor is a nematode which causes an emerging helminthiasis limited to the American Continent, without being a public health problem. Its distribution is neotropical and it was found in the following countries: Brazil, Colombia, Venezuela, Mexico, Costa Rica, Trinidad, Suriname and Bolivia.^1^

This is a case of a patient infested by a nematode of the Lagochilascaris minor genus, located in the middle ear, producing a coalescent mastoid on the right side with a fistulized abscess on the mastoid tip and on the same side of the neck, and this was the first case described in Paraguay.

## CASE REPORT

A 20 year old male, coming from the rural area of Departamento de Itapua, Companhia Santa Ana of the Alto Verá district and with a history of 6 months of evolution, having a growth and pain on the right retroauricular region and on the parotid. He had been having otorrhea for two months with a purulent secretion and right-side retroauricular oozing which alleviated the pain. He reports shedding worms through the retroauricular region and also through the mouth.

Personal background: he mentioned he had eaten wild animals.

In the otorhinolaryngological exam the patient was healthy, he was not running a fever and there was an intense inflammatory process involving the retroauricular region, the parotid and the right submandibular region. There as a fistula on the retroauricular region involving the mastoid tip and on the lateral neck (Photo a). Upon otoscopy we noticed an edematous and stenosed external auditory meatus. The tympanic membrane had a 10mm central perforation and there was purulent secretion oozing from it. He had level II trismus, which made it difficult from him to open his mouth.

**Figure:**
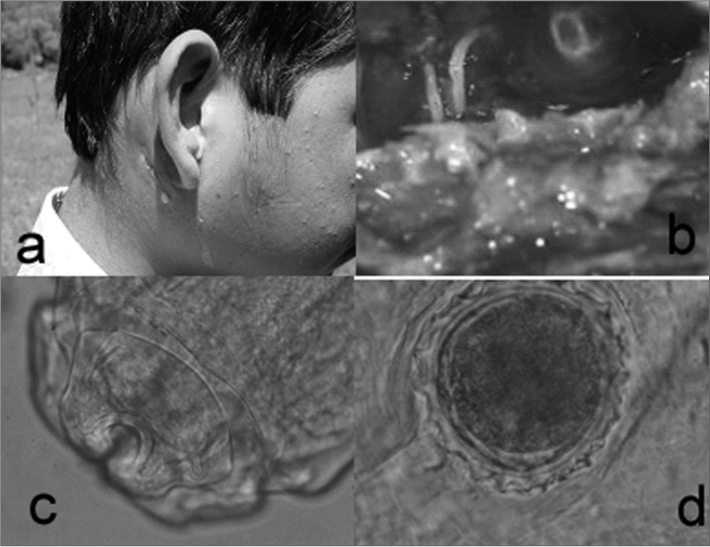
Photo. Lagochilascaris minor – a. Youngster with an inflammatory process on the retroauricular, parotid and submandibular regions. Otorrhea and retroauricular fistula. b. Worms in the mastoid cavity, seen during mastoidectomy. c. Lagochilascaris minor's head showing the three lips. d. Eggs from Lagochilascaris minor.

He was then submitted to a canal wall up mastoidectomy and there was secretion oozing from both lesions and worms in the mastoid cavity (Photo/b).

The material sent for a microbiological study of the worms showed morphological characteristics, such as the lips and the wings, resembling reptile ascarid (Photo/c). We did a serial collection of fecal material which showed beer-bottle-cap-shaped eggs (Photo/d).

The patient remained in the hospital for two weeks and received oral antibiotics (amoxicillin with sulbactam) followed by Ivermectin (200 ug/kg per week) and thiabendazole (1 tab/day for three days, during 15 days). After hospital discharge the patient was instructed to continue with treatment in an outpatient basis.

He had a favorable outcome in the immediate post-op, however slow, with an improvement in the right ear suppuration; nonetheless, the inflammatory process remained in the neck. He also improved on the trismus. The patient did not return on his control visits. Six months after hospital discharge, through the telephone and the Internet we were told the patient had the same manifestation again and was being medicated with anti-parasitic agents.

## DISCUSSION

The Lagochilascaris minor parasitic infestation is the first case described in Paraguay, and it affected a young man from the rural area, in a district in the south of the country, where people have the habit of eating the meat of wild animals; the patient confirmed he had also done so. The transmission mechanism is not fully unveiled. There are doubts as to the host of Lagochilascaris minor or even about its development cycle. The disease is apparently acquired when one eats the meat of contaminated wild animals.^1^, ^2^, ^3^, ^4^

The parasite affected the right-side mastoid, producing a coalescent mastoid and formed a fistulized abscess on the retroauricular and lateral neck. This type of presentation is frequent according to the literature. ^1^,^2^

Diagnosis is defined as the presence of Lagochilascaris minor on the affected region^5^.

The clinical picture is characterized as a chronic process which develops during a period of many months, a differential diagnosis must consider paracoccidioidomycosis, tuberculosis, actinomycosis and leishmaniosis^3^,^4^. In the clinical case hereby presented, besides the microbiological study of the worm, which showed morphological characteristics of adult Lagochilascaris minor, we did a serial collection of fecal material which showed eggs shaped like a beer bottle cap.

The parasite heterogeneous cycle has made treatment hard and today there is no well established treatment scheme which can ultimately treat this disease. It would be necessary to have a drug or pharmacological association which would act on the adult worm and also on the larvae and eggs. Otherwise, the cycle restarts from the resistant forms.

Benzimidazole derivatives associated with Ivermectin, used for a long period of time has yielded good results^4^,^6^.

Unfortunately, the patient did not return for later control visits. Six months after hospital discharge, through a telephone communication, we were informed that the patient had the same clinical manifestation again and was being treated with anti-parasitic agents.

## FINAL REMARKS

Although this is a rare disease, and one which affects a certain range of the rural population who eat the meat of wild animals, otorhinolaryngologists must be attentive to the differential diagnosis of other diseases, especially the granulomatosis.
